# Supplementary figures and images for: Local-scale virome depiction in Medellín, Colombia, supports significant differences between Aedes aegypti and Aedes albopictus
Source: PLoS One. 2022 Jul 27;17(7):e0263143. doi: 10.1371/journal.pone.0263143 (PMC9328524; doi:10.1371/journal.pone.0263143)

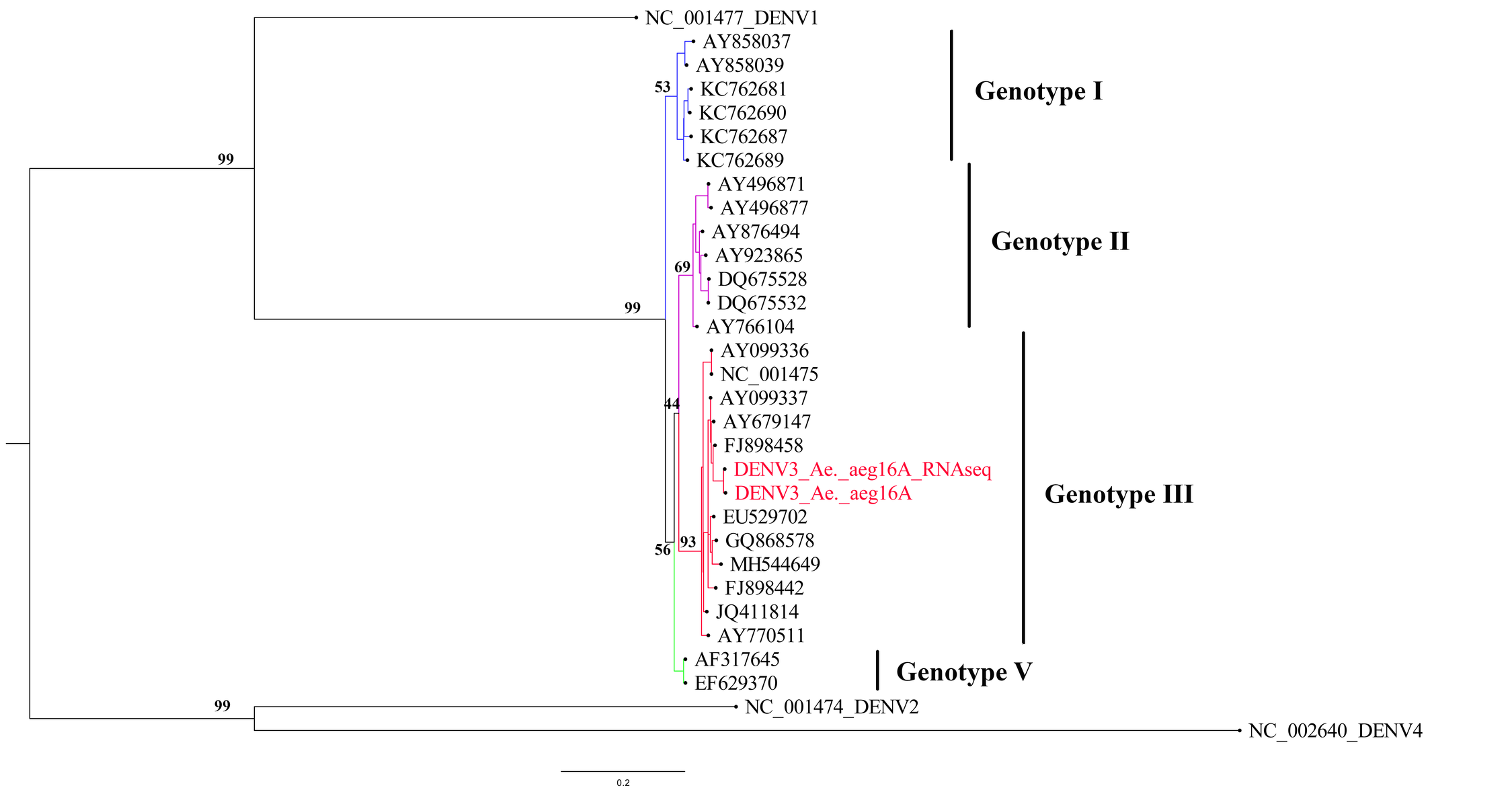

Supplement: S1 Fig — The contig was assembled from RNA-seq data, and the fragment was amplified and sequenced using the Sanger method. The sequences generated in this study are indicated in red. The scale bar indicates the number of substitutions per site. (TIF) [file pone.0263143.s001.tif]
