# Supplementary material for: Local-scale virome depiction in Medellín, Colombia, supports significant differences between Aedes aegypti and Aedes albopictus
Source: PLoS One. 2022 Jul 27;17(7):e0263143. doi: 10.1371/journal.pone.0263143 (PMC9328524; doi:10.1371/journal.pone.0263143)
Supplement: S1 Table — (DOCX) [file pone.0263143.s002.docx]

|  | **Ae. aeg 2015A** | **Ae. aeg 2015B** | **Ae. aeg 2016A** | **Ae. aeg 2016B** | **Ae. aeg 2017A** | **Ae. aeg 2017B** | **Ae. aeg 2018A** | **Ae. aeg 2018B** | **Ae. aeg 2019A** | **Ae. aeg 2019B** | **Ae. alb 2017** | **Ae. alb 2018** | **Ae. alb 2019A** | **Ae. alb 2019B** |
| --- | --- | --- | --- | --- | --- | --- | --- | --- | --- | --- | --- | --- | --- | --- |
| Total Contigs | 36,218 | 37,675 | 182,715 | 246,190 | 32,890 | 249,411 | 192,911 | 208,717 | 239,058 | 284,180 | 201,886 | 190,841 | 211,266 | 205,293 |
| N50 (bp) | 344 | 312 | 354 | 336 | 258 | 349 | 384 | 307 | 382 | 363 | 352 | 385 | 386 | 360 |
| Mean contig length (bp) | 210 | 322 | 288 | 304 | 241 | 365 | 373 | 385 | 396 | 383 | 350 | 375 | 376 | 356 |
| Max contig length (bp) | 12,905 | 3,474 | 5,204 | 5,015 | 3,101 | 4,616 | 5,008 | 6,107 | 6,007 | 5,131 | 6,973 | 6,195 | 5,927 | 5,795 |
| Contigs > = 1 kb | 1,527 | 486 | 2,915 | 2,681 | 68 | 2,527 | 4,471 | 5,676 | 6,234 | 3,400 | 3,122 | 4,450 | 3,551 | 3,534 |

**S1 Table.** Summary of assembly metrics
